# A Novel Polyurethane-Based Polyion Complex Material with Tunable Selectivity against Interferents for Selective Dopamine Determination

**DOI:** 10.3390/bios13060638

**Published:** 2023-06-09

**Authors:** Zixin Zhang, Hongchen Guo, Yuugo Hirai, Katsunori Takeda, Chiho Asai, Naohiro Takamura, Osamu Niwa

**Affiliations:** 1 Advanced Science Research Laboratory, Saitama Institute of Technology, 1690, Fusaiji, Fukaya, Saitama 369-0293, Japan; zixinzhang93@gmail.com; 2 Department of Life Science and Green Chemistry, Graduate School of Engineering, Saitama Institute of Technology, 1690, Fusaiji, Fukaya, Saitama 369-0293, Japan; 3 R&D Headquarters, DKS Co., Ltd., 5 Ogawara, Kisshoin, Minami, Kyoto 601-8391, Japan; ka.takeda@dks-web.co.jp (K.T.); c-asai@dks-web.co.jp (C.A.); n-takamura@dks-web.co.jp (N.T.)

**Keywords:** selective membrane, polyurethane, polyion complex, dopamine detection, biosensor

## Abstract

Polyion complex (PIC) materials have been widely used in biosensors due to their molecular selectivity. However, achieving both widely controllable molecular selectivity and long-term solution stability with traditional PIC materials has been challenging due to the different molecular structures of polycations (poly-C) and polyanions (poly-A). To address this issue, we propose a novel polyurethane (PU)-based PIC material in which the main chains of both poly-A and poly-C are composed of PU structures. In this study, we electrochemically detect dopamine (DA) as the analyte and L-ascorbic acid (AA) and uric acid (UA) as the interferents to evaluate the selective property of our material. The results show that AA and UA are significantly eliminated, while DA can be detected with a high sensitivity and selectivity. Moreover, we successfully tune the sensitivity and selectivity by changing the poly-A and poly-C ratios and adding nonionic polyurethane. These excellent results were employed in the development of a highly selective DA biosensor with a detection range from 500 nM to 100 μM and a 3.4 μM detection limit. Overall, our novel PIC-modified electrode has the potential to advance biosensing technologies for molecular detection.

## 1. Introduction

Polyion complex (PIC) membranes are mixtures of oppositely charged polymer compounds [1,2]. This material has been widely studied and applied in the fields of drug delivery, batteries, fuel cells, and biosensors due to its unique structure and selectivity [3,4,5,6,7,8,9]. For example, Raveendran et al. developed an estrone-appended PIC micelle to deliver cytotoxic peptide to breast cancer cells [7]; Lee et al. proposed a hybrid PIC micelle to achieve a high-performance lithium battery [8]; and Nagar et al. synthesized a PIC membrane that can be employed as a solid polymer electrolyte for direct methanol fuel cell application [9].

In terms of biosensor applications, PIC materials are widely studied and constructed as oxidase-based biosensors, as shown in Table 1. Ballesta-Claver et al. constructed a highly sensitive lactate chemiluminescent biosensor using poly-L-lysine (PLL) and a poly(4-styrenesulfonate) (PSS)-based PIC material as a lactate oxidase immobilizer [10]. The PLL and PSS-based PIC material was thoroughly studied by Mizutani’s group [11,12,13]. They reported the molecule-sieve effect of a PIC membrane, where the electrochemical signal decreases sharply when the molecular weight (Mw) of the analyte exceeds 100 [11]. This result is undoubtedly beneficial for constructing highly selective and accurate biosensors because common interferents in body fluids such as L-ascorbic acid (Mw = 176.12) and uric acid (Mw = 168.11) can be suppressed using the above PIC materials. However, the use of PLL-PSS PIC membranes cannot sensitively detect biomarkers with molecular weights greater than 100, such as glucose (Mw = 180.156) and dopamine (Mw = 153.18). To overcome this dilemma, Mizutani et al. proposed a bilayer structure for an enzyme/PIC-modified electrode for detecting analytes whose molecular weights exceed 100 [12,13].

Thanks to these instructive results, the PLL-based PIC can be successfully applied to the field of biosensors. For example, a D-amino acid biosensor was proposed by Yabuki et al. [14], and a glucose sensor was developed by Katsuno et al. [15]. The above biosensor with an enzyme can solve the permeation problem posed by larger Mw analytes because the Mw of electroactive species produced by the enzymatic reaction is usually smaller than that of their substrates, and so can easily permeate the PIC membrane. However, the enzyme layer is not stable because it is not protected by any membranes such as PIC. Moreover, it is very difficult to construct a second-generation enzymatic biosensor and a third-generation DET biosensor because the enzyme layer should be modified on a conventional PIC layer, which means the enzyme layer is separate from the electrode surface. 

To overcome the above problem, widely controllable molecular permeation by PIC is necessary, which could be achieved by changing the interaction between the polycation (poly-C) and the polyanion (poly-A) in PIC. One method of controlling the interaction between poly-C and poly-A is to change the ratio of both polyions. However, the PIC will be more unstable if the poly-C/poly-A ratio is significantly unbalanced, since the stability of the previously reported PIC is only maintained by an electrostatic interaction, and the main chains of poly-C and poly-A are totally different. The compatibility and stability of both polyions should be improved if their main chain is similar.

Recently, a polyurethane (PU) and epoxy-enhanced polyurethane (E-PU) biocompatible material was reported for a needle-type in vivo glucose biosensor [16]. This biosensor is formed with a Pt/polyaniline/GOx/PU/E-PU multi-layer structure. The electropolymerized polyaniline membrane supports a porosity nanostructure for enzyme immobilization, and the PU and E-PU membranes are coated separately on a metal wire. As a result, this multilayer-structure biosensor provides long-term 26-day stability and an accurate result for the in vivo biosensing of glucose. After optimization, the detection range of this biosensor is from 0 to 10 mM in simulated human serum samples, with an in vivo sensitivity of 1.34 μA/mM×cm^−2^. Although polyurethane represents an ideal candidate for a biocompatible biosensor membrane suitable for continuous measurement, widely controlled selectivity was not studied in this report. In addition, electropolymerized polyaniline, which has a different structure from that of polyurethane, is required, and this increases the number of modification steps.

**Table 1:** Recent PIC constructed biosensors.

PIC Materials		Analyte	Molecular Weight	Sensitivity (nA/μM×cm^2^)	Reference
Polyanion	Polycation				
DNA	Poly-L-lysine (PLL)	H_2_O_2_	34	6.6 ± 0.3~7.1 ± 0.3	[17]
		L-Ascorbic acid	176	5.1 ± 0.9~11.1 ± 0.9	
		Urate	168	6.3 ± 0.9~15.1 ± 1.1	
		Dopamine	158	4.2 ± 0.4~7.6 ± 1	
		4-Acetaminophen	151	23.4 ± 1.7~26.3 ± 2	
DNA-Cu (II)	Poly(allylamine) hydrochloride (PAA)	H_2_O_2_	34	0.03 ~45.8	[18]
Poly(sodium-4-styrenesulfonate) (PSS)	PLL	Urea	60	1.7	[19]
Poly(acrylic acid)	PLL	Glucose	180	Not Given	[15]
Poly(acrylic acid)	PAA				
PSS	PLL	Glutamate	147	20	[13]
		Lactate (chemiluminescent biosensor)	90	Not Given	[10]
Polyurethane-based anion	Polyurethane-based cation	Dopamine	158	68.9~160.1	This work
		L-Ascorbic acid	176	14.1~203.2	
		Uric acid	168	6.9~85.1	

Here, we first propose a waterborne novel PU-based PIC material, including the poly-A type, poly-C type, and nonionic type. We expected to realize widely tunable selective properties by changing the ratios of the above three kinds of waterborne PUs by using dopamine (DA) as the analyte and using L-ascorbic acid (AA) and uric acid (UA) as interferents. We also studied the interaction between nonionic PU and poly-A and poly-C. A low detection limit below the μM level with a highly selective detection result for DA was achieved by utilizing an optimized biosensor. The sensitivities of electroactive species at previously reported PIC and PU-based PIC-membrane-modified electrodes are collected in Table 1. The mechanism of molecular selectivity between previous reported PIC materials and our PU-based PIC was thoroughly discussed. Finally, we examined the anti-common interferent performance of our PIC by using PIC modified-GC electrodes to detect DA in the presence of UA, AA, and 3,4-dihydroxyphenylacetic acid (DOPAC).

## 2. Materials and Methods

### 2.1. Reagents and Materials

The poly-A (35 wt%; concentration of anion functional group: 0.75 mM), poly-C (26 wt%; concentration of cation functional group: 0.48 mM), and nonionic PU were synthesized in the application laboratory of DKS Co., Ltd. (Kyoto, Japan), and the synthesis method was similar to previous reports [20,21].

Dopamine (DA), 3,4-dihydroxyphenylacetic acid (DOPAC), uric acid (UA), and L-ascorbic acid (AA) were purchased from Fujifilm (Osaka, Japan). A solution of 0.1 M PBS buffer (pH = 7.4) was used to prepare DA, UA, AA, and DOPAC solutions for electrochemical measurements. 

### 2.2. Preparation of PIC Membrane Modified-Electrodes

**Figure 1:**
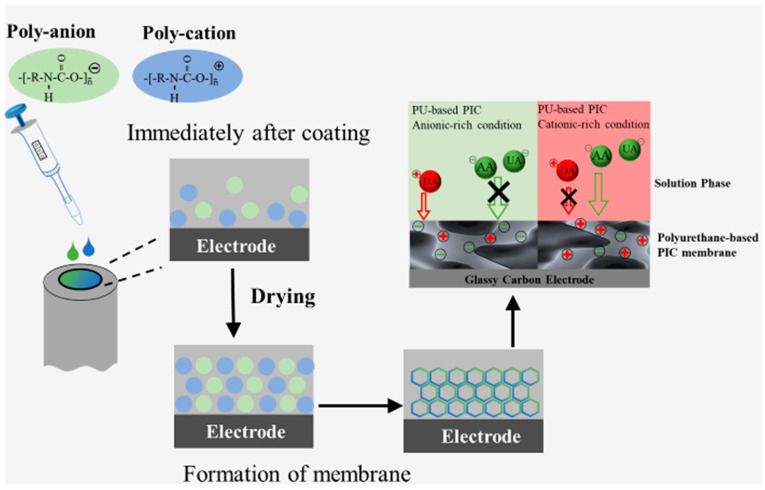
Schematic diagram of PU-based PIC membrane preparation.

Figure 1 shows a schematic diagram of the novel PU-based PIC membrane preparation method. The membrane was prepared by dropping poly-A and poly-C solutions after 10-fold (20-fold, or 30-fold) dilution and mixing them on GC electrodes. Then, the PIC-modified electrodes were dried in an incubator at 30 °C for 2 h.

PIC film thickness is measured by using a thin glass plate to measure PIC film thickness instead of GCE. We prepared PIC films consisting of different ratios of poly-A and poly-C (and nonionic PU solution) dropwise onto the glass substrate by adjusting the surface area to that on the GCE. After drying it in an incubator at a constant 30 °C for 2 h, a commercially available digital micrometer was then used to calculate the thickness of the different PIC films (*n* = 3).

### 2.3. Apparatus

An electrochemical analyzer (ALS model 1220C BAS Co., Ltd., Tokyo, Japan) was used for all the electrochemical measurements including constant potential amperometry (i-t) and cyclic voltammetry (CV). A GC electrode (d-1.6 mm BAS Co., Ltd., Tokyo, Japan), Ag/AgCl electrode (RE-3V BAS Co., Ltd., Tokyo, Japan), and Pt wire were used as working, reference, and counter electrodes, respectively.

### 2.4. Electrochemical Analysis

For constant i-t measurements, the potential of the GC and PIC-modified GC electrodes was set at 0.5 V vs. Ag/AgCl. During the i-t measurements, each sample solution (DA, L-AA, UA, or DOPAC) was injected into a 0.1 M PB buffer (pH = 7.4) solution while stirring with a magnetic bar.

## 3. Result and Discussion

### 3.1. Selectivity of PU-Based PIC-Membrane-Modified Electrodes

Polymer-modified electrodes have been used to construct highly selective biosensors as a result of their molecule cut-off property. For example, Mizutani et al. used a PLL-PSS-based PIC membrane with an Mw cut-off property of 100, which was used to determine L-lactate in human serum and yoghurt [11]. Kastuno et al. constructed a highly selective bi-layered glucose enzymatic biosensor by utilizing the Mw cut-off property differences between PAm-PAA and PLL-PAA membranes [15]. However, the above works did not study the relationship between the sensitivity of the sensor and the mixing ratios of poly-A/poly-C. It might be difficult to obtain the stable PIC by greatly changing the mixing ratios of poly-A and poly-C and control Mw cut-off property using traditional PIC materials, because the main-chain structures of both polyions are different.

Here, we developed a PIC membrane consisting of synthesized waterborne PU-based poly-A and poly-C, whose properties can be changed by changing the quantity of charged groups in the membranes. In this section, we determine the selectivity of our PIC membranes by measuring dopamine as the analyte and AA and UA as the interferents. This is because DA is a commonly detected neurotransmitter, and AA and UA are common interferents in body fluids.

**Figure 2:**
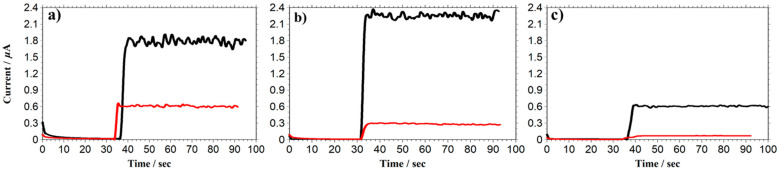
Responses of DA, AA, and UA at PU-based PIC-modified GC electrodes. (**a**) 72 μM DA (Mw = 168.1), (**b**) 72 μM AA (Mw = 153.2); (**c**) 72 μM UA (Mw = 197.2). Red plot: PIC-modified GC electrode; black plot: GC electrode; poly-A: poly-C = 2:1; applied potential: +0.5 V vs. Ag/AgCl; electrolyte: 0.1 M PB buffer, pH = 7.4.

Figure 2 shows the selective property of GC electrodes modified and unmodified with a PU-based PIC membrane by comparing the amperometric responses of DA, AA, and UA in a 0.1 M PB buffer (pH 7.4). The purpose of this study for evaluating the selectivity of a PU-based PIC membrane by changing the ratio of each polyion. For such purposes, the suitable concentration is needed. When too high a concentration is used, larger than 1 mM, the responses of each species will be slower, since the electron transfer of DA and interferents is not as high as those of typical redox species such as ferricyanide. In contrast, much lower concentrations such as physiological-level concentrations create difficulty in evaluating the selectivity accurately since the background noise level cannot be negligible for amperometry and the capacitive current is a problem for CV measurement. By taking account of the above problems, we chose concentrations of each species of 72 μM to evaluate the selectivity of PIC films accurately. The ratio of poly-A to poly-C is 2:1, and the quantities of anionic and cationic functional groups modified on the electrode were 0.30 nmol/electrode and 0.09 nmol/electrode, respectively. Therefore, the excessive anionic functional group amounts to 0.21 nmol/electrode.

The responses of DA, AA, and UA decreased by about 65.7%, 87.3%, and 85.6%, respectively, at GC electrodes modified with PIC compared to those of the unmodified electrode in Figure 2. Although the currents of all species decreased due to the slower diffusion coefficient in the PIC membrane, the DA response is higher than those of AA and UA. This is due to electrostatic repulsion between excessive anionic functional groups in the PIC membrane and anionic-charged AA and UA, which significantly suppressed the AA and UA diffusion process. In contrast, the DA response is not greatly decreased compared with those of AA and UA in Figure 2, suggesting that cationic-charged DA is attracted by anionic functional groups in the PIC membrane.

Such selectivity differs from the previously reported PIC materials because the selectivity mechanism of traditional PIC materials is caused by their molecule cut-off property. With an Mw cut-off property of less than 100, it is difficult to detect DA (Mw = 168.1); furthermore, glucose (Mw = 180.2), which is the most important and commonly detected analyte, is difficult to detect with a high sensitivity using an electrochemical biosensor.

Thanks to the different selective mechanism of our PIC, a higher selectivity in the presence of a similar Mw level of interferents can be achieved compared with the previously reported traditional PIC materials shown in Table 1. The sensitivity of electroactive species with an Mw exceeding 100 at a PU-based PIC modified electrode is one to two orders higher than those at electrodes modified with traditional PIC materials. This might be because the main chain of the PU-based PIC is the similar, allowing the polymer structure to remain more stable even when the poly-A and poly C ratio is greatly changed. In contrast, with traditional PIC materials, the ratio of poly-A and poly-C cannot change freely due to the different polymer main chains [11,14,15].

The relationship between the selectivity and the excessive functional group must be optimized, since the balance between poly-A and poly-C affects not only selectivity but also sensitivity. This is because the quantity of excessive functional groups will affect the density of PIC, which greatly changes the diffusion of molecules.

### 3.2. Study of the Effect of Excessive Functional Groups on PIC Selectivity

**Figure 3:**
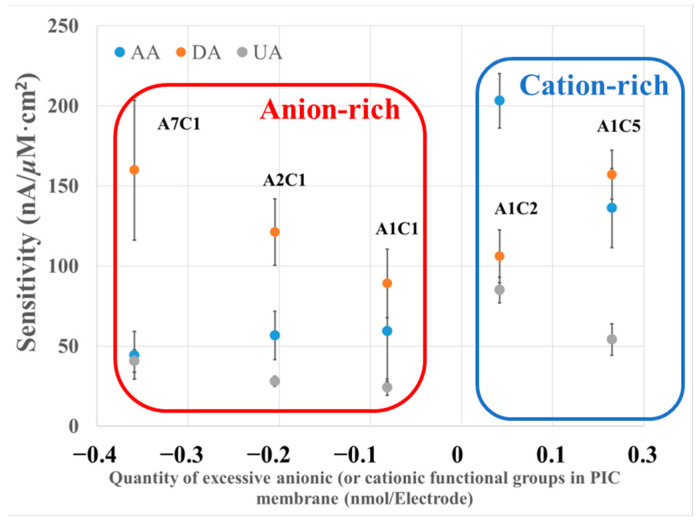
The relationship between the quantity of excessive functional groups in the PIC membrane and the responses of the analyte and interferents. The concentration of DA, AA, and UA is 72 μM; applied potential: +0.5 V vs. Ag/AgCl; electrolyte: 0.1 M PB buffer, pH = 7.4; *n* = 3.

We studied the selectivity of PIC by widely changing the poly-A and poly-C ratios to understand the effect of PIC composition on sensitivity and selectivity. The current of each species at the PIC-modified electrodes with different poly-A/poly-C ratios is shown in Figure 3. The relative sensitivities of UA and AA normalized by the oxidation current of DA are shown in Table 2. The ratios of poly-A to poly-C were 7:1, 2:1, 1:1, 1:2, and 1:5, and the PIC modified-GC electrodes with different ratios were simplified to A7C1, A2C1, etc. The quantity of excessive functional groups and the selectivity of each analyte were calculated with the following Equations (1) and (2).

(1) $$c_{excessive\;\;}=-c_{anionic\;}+c_{cationic\;}$$

(2) $$Sensitivity=i_{p}÷c_{analyte\left(or\;interferent\right)}$$

where *c_excessive_* is the quantity of excessive charged groups, and *c_anionic_* and *c_cationic_* are the quantities of anionic and cationic functional groups on the electrode, respectively. *i_p_* is the amperometric current, and *c_analyte(or interferent)_* is the concentration of each species.

The sensitivity of DA on PU-modified GC electrodes was observed from 371.4 to 560.0 nA/μM×cm^2^ as shown in Figure 3.

When the ratio of poly-A/poly-C was greatly changed, the sensitivity of DA increased, but the error magnitude was relatively large under the condition of A7C1. When the ratio of poly-A/poly-C was highly imbalanced, the structure of the PIC membrane loosened due to the weaker interaction between poly-A and poly-C, which decreased the density of the membrane and increased the diffusion of DA. 

In contrast, both the sensitivity and selectivity of AA and UA decreased as the quantity of the excess anionic group increased, as shown in Figure 3 and Table 2. Specifically, for AA in an anion-rich condition, the sensitivity was determined to be from 155.1 to 356.0 nA/μM×cm^2^, approximately 0.5 or 0.3 times that of DA, which suggested the selective detection of DA under an anion-rich condition. In contrast, under a cation-rich condition, the sensitivity of AA was determined to be from 476.9 to 711.3 nA/μM×cm^2^, which is approximately equivalent to or even higher than that of DA. This result suggests that the electrochemical responses of AA and UA were suppressed by anionic-charged groups and improved by cationic-charged groups. 

As summarized above, we studied the selectivity of our PU-based PIC materials. The selectivity is highly tunable by varying the poly-A/poly-C ratio, which realized selectivity for biomolecules with a similar molecular weight, unlike previously reported PIC-modified electrodes. As a result, it is possible to construct a sensitive and selective DA biosensor by optimizing poly-A and poly-C ratios similar to the A2C1 membrane. This result is undoubtedly beneficial for constructing various highly selective and sensitive biosensors.

### 3.3. Optimization of PIC Thickness and Density for DA Biosensor Selectivity

To optimize the sensitivity and selectivity of the DA biosensors, we first studied the effect of membrane thickness on the electrochemical response of DA, AA, and UA using A2C1-modified electrodes. This is because the fluctuation of the current at an A7C1-modified electrode is large compared with those with A2C1-modified electrodes, although the sensitivity of DA on A2C1 is lower than that on A7C1.

**Figure 4:**
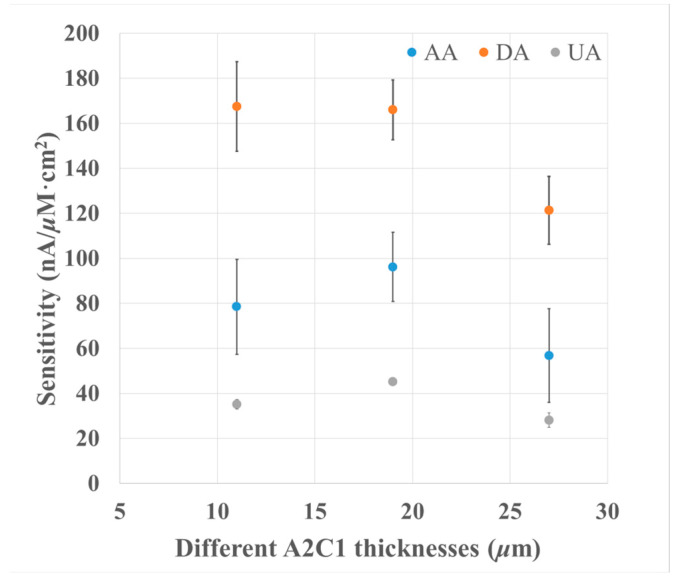
The relationship between the thickness of the A2C1 membrane and the selectivity responses of the analyte and interferents. The DA, AA, and UA concentration is 72 μM; applied potential: +0.5 V vs. Ag/AgCl; electrolyte: 0.1 M PB buffer, pH = 7.4; *n* = 3.

Figure 4 shows the sensitivities of DA, AA, and UA at different dilution folds of A2C1-modified electrodes, and the normalized sensitivities are shown in Table 3. Dilution folds of 10, 20, and 30 were chosen for poly-A and poly-C, and the thickness of each membrane was 27, 19, and 11 μm, respectively. The decrease in A2C1 thickness from 27 to 11 μm resulted in an increase in the sensitivities of DA, AA, and UA by 37.9% (from 425 to 586 nA/μM×cm^2^), 38.2% (from 199 to 275 nA/μM×cm^2^), and 25% (from 98.4 to 123 nA/μM×cm^2^), respectively. Moreover, similar normalized sensitivity results for AA and UA can be seen in Table 3. These results suggest that decreasing the membrane thickness only enhances the sensitivities of DA, AA, and UA due to the increase in the diffusion of analytes and interferents. 

However, despite the thickness decrease, the anionic-charged groups in the PIC membrane still inhibited the incorporation of AA and UA with the PIC membrane, because the PIC membrane has a homogeneous structure. Therefore, a reduction in membrane thickness only contributes to an increase in sensitivity, but does not increase the selectivity of DA. 

To further improve the selectivity, we focused on an A7C1-membrane-modified electrode. This is because the selectivity of DA against AA and UA is potentially high, as shown in Figure 3, although the data fluctuation is somewhat large due to the weaker interaction between poly-A and poly-C caused by a large anion and cation difference. We attempted to address this by adding nonionic waterborne PU to enable us to prepare a PIC membrane to modify repulsion through an excess quantity of anions.

**Figure 5:**
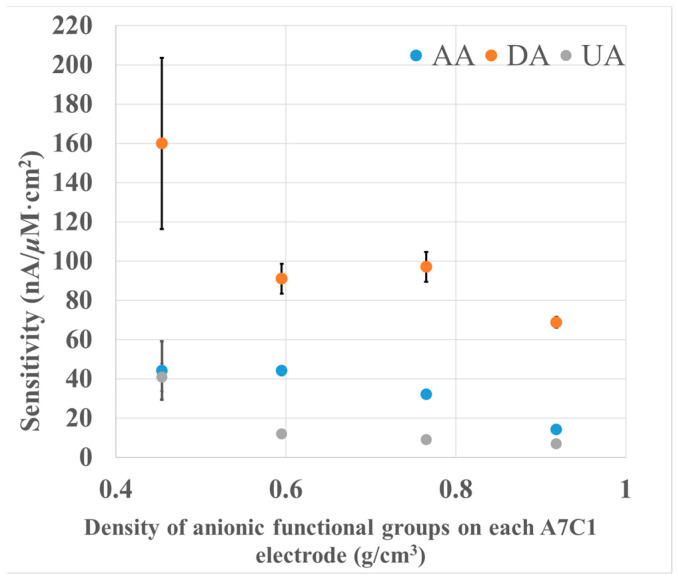
Relationship between the thickness of the A7C1 membrane and the selectivity responses of the analyte and interferents. The concentration of DA, AA, and UA is 72 μM; applied potential: +0.5 V vs. Ag/AgCl; electrolyte: 0.1 M PB buffer, pH = 7.4; *n* = 3.

Figure 5 shows the sensitivities of AA and UA at the A7C1 (after 20-fold dilution) modified electrode after adding different amounts of nonionic PU. The normalized sensitivities are shown in Table 4.

Although the sensitivities of DA, AA, and UA decreased as the amount of nonionic PU increased, the selectivity of DA increased by 5 times and 10 times against AA and UA, respectively, as shown in Figure 5 and Table 4. One reason for the decrease in the sensitivities of DA, AA, and UA is the increased membrane thickness. However, the membrane thickness does not affect the selectivity as already shown in Figure 4 and Table 3. It should be noted that the selectivity of DA against AA and UA was improved by increasing the A7C1 thickness by adding nonionic PU. This difference might be attributed to the increased polymer density (from 0.60 to 0.92 g/cm^3^) caused by the mixing of nonionic PU, as shown in Table 4. 

To better illustrate the effect of the addition of nonionic PU to poly-A and poly-C, we compared the selectivity of two PIC membranes with similar thicknesses. One was A2C1 after 10-fold dilution (A2C1-10-fold), whose thickness and density were 27 μm and 1.11 g/cm^3^, respectively. The other was A7C1 after 20-fold dilution and mixing with 1 μL nonionic PU (A7C1 + 1 μL), whose thickness and density were 28 μm and 0.77 g/cm^3^, respectively. In spite of the lower density, the latter membrane showed a better selectivity of DA against AA and UA, indicating that nonionic PU modifies the repulsion of excess anionic groups in the PIC membrane and improves the interaction of poly-A and poly-C, which might stabilize the PIC membrane in spite of the existence of highly excess anionic groups.

In addition, a significant decrease in the error was observed by adding the nonionic PU as shown in Figure 5 compared with the A7C1 membrane without nonionic PU.

Since the selectivity of DA increased by increasing the amount of nonionic PU, we chose a membrane consisting of A7C1 after 20-fold dilution and 2 μL nonionic PU as the optimal condition.

## 4. Dopamine Calibration Curves

**Figure 6:**
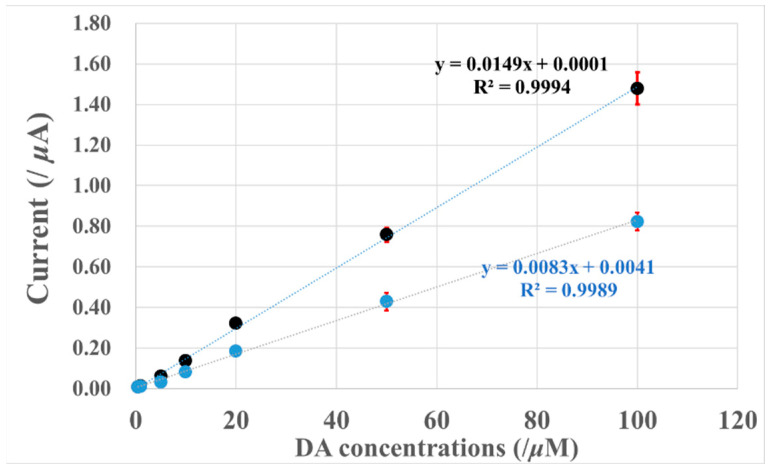
Calibration curves of oxidation currents at different electrodes against DA concentration. Black: A2C1-20-fold electrode; blue: A7C1 + 2 μL electrode; applied potential: +0.5 V vs. Ag/AgCl; electrolyte: 0.1 M PB buffer, pH = 7.4; *n* = 3.

Figure 6 shows the DA calibration curves obtained at 0.5 V (vs Ag/AgCl) with A2C1- and A7C1-membrane-modified electrodes. In both membranes, the DA selectivity was optimized.

The linear-regression equations of each PIC-modified GC electrode showed good linear relationships from 500 nM to 100 μM. The detection limits were calculated to be 2.4 μM and 3.4 μM (S/N = 3) for A2C1-20-fold and A7C1-20-fold + 2 μL PU modified electrodes, respectively. 

Although the sensitivity of DA observed on the A2C1-20-fold electrode is about two times that of the A7C1 + 2 μL PU modified electrode, the detection limits for the two PIC membranes do not differ greatly. Therefore, an A7C1 + 2 μL PU modified electrode might be better for biological sample applications, since the selectivities of DA against AA and UA are about two and three times higher than those of the A2C1-20-fold modified electrode.

## 5. Selectivity of Dopamine Biosensors with Interferents

**Figure 7:**
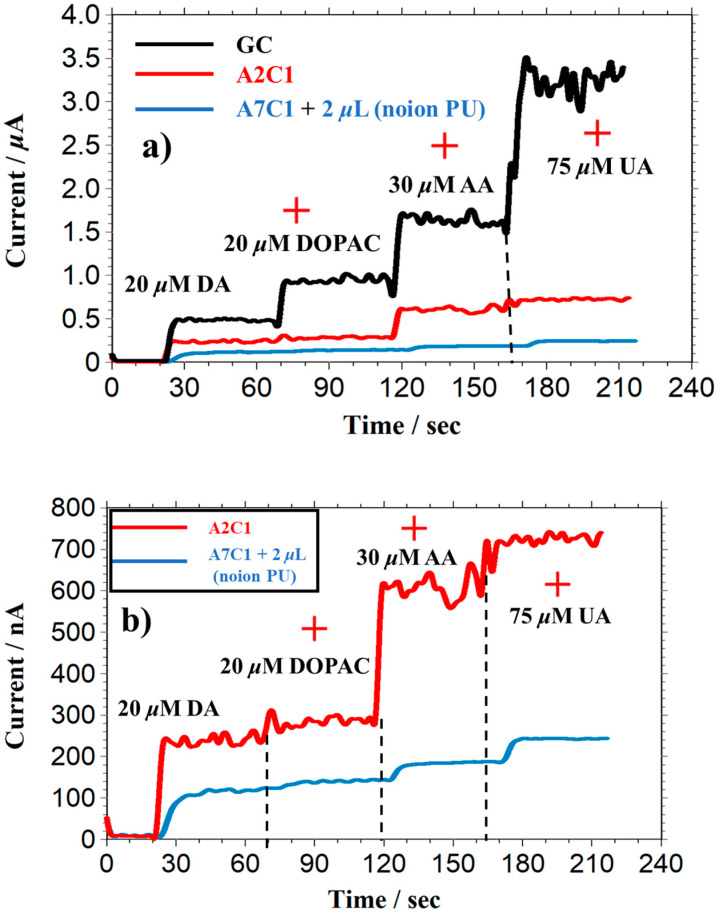
I-t curve of successive addition of DA, UA, AA, and DOPAC on different electrodes. (**a**) GC, A2C1, and A7C1 (+2 μL no-ion PU) electrodes; (**b**) A2C1 and A7C1 (+2 μL no-ion PU) electrodes. Applied potential: +0.5 V vs. Ag/AgCl; electrolyte: 0.1 M PB buffer pH = 7.4; initial electrolyte volume: 15 mL.

Continuous amperometric measurements of DA were performed by adding interferents to examine the selectivity of the A2C1-20-fold and A7C1 + 2 μL membrane-modified electrodes, as shown in Figure 7. In these measurements, we evaluated the effect of DOPAC, because DOPAC is a metabolite of DA in the human body and has a similar chemical structure to that of DA.

As expected, the responses of interferents were significantly suppressed on both A2C1-20 fold and A7C1 + 2 μL modified electrodes compared with bare GC electrodes. In particular, with the latter electrode, the electrochemical responses of DOPAC, AA, and UA were almost eliminated, which is consistent with the data presented in Table 4. 

**Figure A1:**
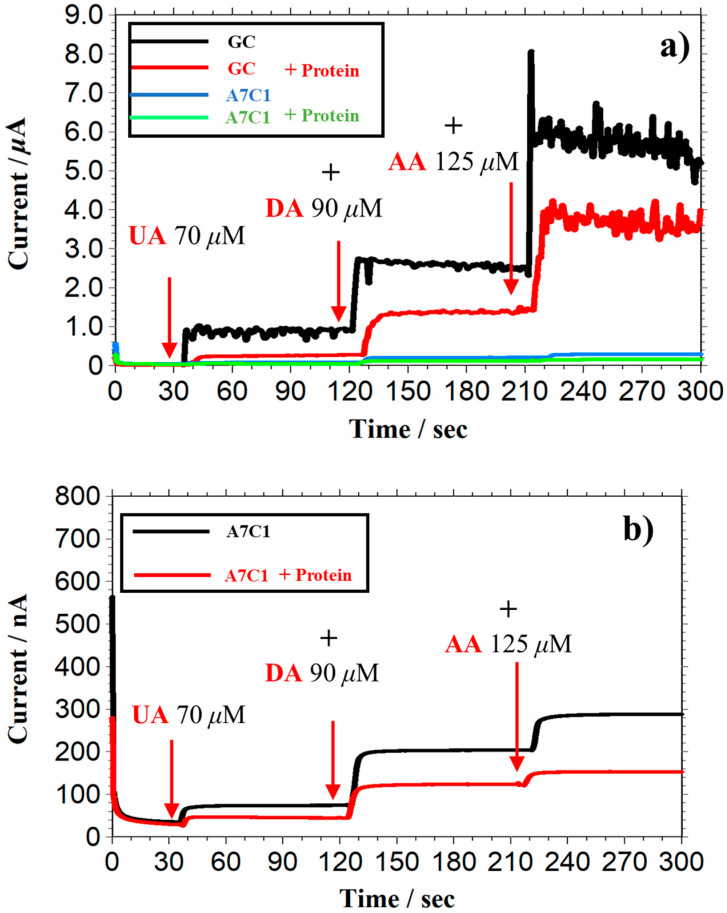
I-t curve of bare and different modified GC electrodes upon successive addition of UA, DA, and AA in protein-free and protein-containing PB buffer. (**a**) Bare and A2C1-modified GC electrode. (**b**) A2C1 and A7C1 (+2 μL no-ion PU)-modified GC electrode. (Applied potential: +0.5 V vs. Ag/AgCl; electrolyte: 0.1 M PB buffer pH = 7.4; initial electrolyte volume: 15 mL; concentrations of protein: ABL: 1.6 mg/mL + GLB: 1.4 mg/mL).

In addition, the simulated real experiments were also performed as shown in Figure A1 and the data collected in Table 5. As shown in Table 5, by calculating the normalized dopamine against each interferent, the selectivity of AA increased from 1.08 to 1.12, and the selectivity of UA decreased from 0.49 to 0.29, when observed on GC electrode in solutions with and without protein. This might be because the protein adsorbed on the bare GC, reducing the sensitivity of each species. In contrast, the selectivity of AA and UA observed on the A7C1 (+2 μL no-ion PU) electrode decreased from 0.27 to 0.19, and from 0.14 to 0.12 in the solutions with and without protein, respectively. Such similar sensitivity and selectivity with and without proteins indicated that the A7C1 (+2 μL no-ion PU) electrode could achieve a highly selective detection in the solution with proteins. 

The above results indicate that our PIC membrane is a promising candidate for practical biosensing applications such as in vivo detection.

## 6. Conclusions

We have successfully demonstrated the performance of a synthesized waterborne PU-based PIC membrane for highly selective DA detection as described below.

Firstly, the PIC membrane can be prepared by greatly changing the ratios of poly-A to poly-C, which significantly modifies the sensitivity of DA and its selectivity against AA and UA.

Secondly, the selectivity of a PU-based PIC membrane is due to electrostatic repulsion caused by the excess charged functional groups in the PIC membrane, which is a different mechanism from that of previously reported traditional PIC materials.

Thirdly, the selectivity of a PU-based PIC membrane can be tuned by adding a nonionic PU solution, which eases the repulsion between excess charges in PIC when the poly-A and poly-C ratios are very different.

As a result, we could detect DA with a detection limit of 3.4 μM and a linear detection range of 50 nM to 100 μM with little interference from AA and UA using an optimized PIC membrane consisting of A7C1 by adding 2 μL of nonionic PU.

These findings demonstrate that our synthesized PU-based PIC material can be used in various biosensing applications, and specifically for detecting molecules when the analyte and interferents have similar-sized molecules.

## Figures and Tables

**Table 2:** The selectivity of dopamine against each interferent at different poly-A and poly-C ratios. (*n* = 3).

Electrodes	Excessive Functional Group (nM/Electrode)	Normalized Sensitivity (Each Species Selectivity/DA Selectivity)		
		AA	DA	UA
A7C1	−0.36	0.28	1.00	0.25
A2C1	−0.21	0.47	1.00	0.23
A1C1	−0.08	0.67	1.00	0.27
A1C2	0.04	1.92	1.00	0.80
A1C5	0.14	0.87	1.00	0.34
GC		1.27	1.00	0.35

**Table 3:** The selectivity of dopamine against each interferent at different poly-A and poly-C dilution folds (*n* = 3).

Electrodes	Anionic Functional Group Density (nM/mm^3^·Electrode)	Thickness of Membrane (μm)	Normalized Sensitivity (Each Species Selectivity/DA Selectivity)		
			AA	DA	UA
A2C1-10-fold	1.11	27	0.47	1.00	0.23
A2C1-20-fold	0.77	19	0.58	1.00	0.27
A2C1-30-fold	0.89	11	0.47	1.00	0.21

**Table 4:** The selectivity of dopamine against each interferent with different quantities of nonionic PU solution (*n* = 3).

Electrodes	Polymer Density (g/cm^3^)	Thickness of Membrane (μm)	Normalized Sensitivity (Each Species Selectivity/DA Selectivity)		
			AA	DA	UA
A7C1	0.57	18	0.28	1	0.25
A7C1 + 0.5 μL	0.60	24	0.48	1	0.13
A7C1 + 1 μL	0.77	28	0.33	1	0.09
A7C1 + 2 μL	0.92	35	0.21	1	0.10

**Table 5:** The selectivity of dopamine against each interferent with different electrodes in the simulated samples. *n* = 2; Concentrations of protein: ALB: 1.6 mg/mL + GLB: 1.4 mg/mL.

Electrodes	Electrolyte	Normalized Sensitivity (Each Species Selectivity/DA Selectivity)		
		AA	DA	UA
GC	Protein-free	1.08	1.00	0.43
	Protein-containing	1.12	1.00	0.29
A7C1 + 2 μL no-ion PU	Protein-free	0.27	1.00	0.14
	Protein-containing	0.19	1.00	0.12

## Data Availability

The data presented in this study are available on request from the corresponding author. The data are not publicly available due to the research project has not yet concluded.
